# The Origin and Progress of Renal Surgery

**Published:** 1899-05

**Authors:** 


THE ORIGIN AND PROGRESS OF RENAL SURGERY. With special reference to Stone in the Kidney and Ureter, and to the Surgical Treatment of Calculus Anuria; being the Hunterian Lectures for 1898, together with a Critical Examination of Subparietal Injuries of the Ureter. By HENRY MORRIS, M. A., M. B. Lond.; F. R. C. S.; Senior Surgeon to the Middlesex Hospital; Examiner in Surgery in the University of London; Member of the Council and Chairman of the Court of Examiners of the Royal College of Surgeons of England; Hunterian Professor of Surgery and Pathology, Royal College of Surgeons of England; Honorary Member of the Medical Society of the County of New York. Philadelphia: P. Blakiston's Son & Co. 1898.

This collection of lectures is a most valuable acquisition to surgical literature. There is no straining for effect in the work. It is alike of interest to the general practitioner and the specialist.

The book is made up of four lectures as follows: I. On the origin and progress of renal surgery and the conservative tendency of its recent development. II. Renal calculus: The difficulties and errors in diagnosis in their relation to exploration of the kidney--unsuspected, quiescent and migratory calculi. III. Fistula, caused by renal calculus; obstructive anuria, due to renal calculus; the technique of the exploration of the kindey and ureter for calculus. IV. Injuries of the ureter.

Added to these lectures are eleven sets of tables, covering 267 operations performed on the kidney by Mr. Morris and a number of operations for calculus anuria. This combination, historical, tabular and operative, of facts relative to the kidney makes the work especially valuable as a reference. In these days of ambidextrous surgery we must keep well apace with all branches and no recent work so completely and yet so briefly covers the field as this. Not only does Mr. Morris tell in simple, interesting language how to diagnose, but he goes on and tells just as simply how to operate, the lectures being splendidly illustrated.

A considerable part of the third lecture is devoted to calculus anuria. In view of the fact that this type of anuria is too seldom recognised and so little known, Mr. Morris has performed a distinct service to the profession in dealing so extensively with it. The advantages which the surgical treatment affords are so conclusively shown that one naturally wonders why it has not formed the subject of special effort long before this.

After briefly and entertainingly sketching the history of renal surgery from the date of its first known discussion in 1581, by Francois Rousset, up to the present time, the author describes operations for ureterotomy, resection, ureterectomy, anastomoses and grafting. He does not confine himself to his own methods by any means, but illustrates and describes the operations of other eminent surgeons and allows the reader to judge for himself as to which is best.

A great deal of information is given in the sections devoted to etiology, pathology and treatment of calculus, and the technique for operations on the kidney and ureters. The latter subject is so clearly set forth and so admirably handled that it is easily grasped and clearly understood. Mr. Morris has the happy faculty of making himself understood, a gift too infrequently possessed by writers on special subjects. He impresses the reader from the very first and holds attention to the end. Even the tabulations, usually such caviare affairs, with a sort of a hair tonic taste, are interesting because they show results in such a manner as to arouse interest. More such works on special surgical subjects would make the reading-life of the student less weedy.

